# Palm vitamin E reduces locomotor dysfunction and morphological changes induced by spinal cord injury and protects against oxidative damage

**DOI:** 10.1038/s41598-017-14765-3

**Published:** 2017-10-30

**Authors:** Parastoo Mojtahed Zadeh-Ardabili, Sima Kianpour Rad, Soheila Kianpour Rad, Huzwah Khazaài, Junedah Sanusi, Musa-al-Reza Haji Zadeh

**Affiliations:** 1 Anatomy Department, Faculty of Medicine, University of Malaya, 50603 Kuala Lumpur, Malaysia; 2 Molecular Medicine Department, Faculty of Medicine, University of Malaya, 50603 Kuala Lumpur, Malaysia; 3 Faculty of medicine, University of Medical Science, Qazvin, Iran; 4 Physiology department, Faculty of Medicine, University of Medical Science, Mashhad, Iran

## Abstract

Spinal cord injury (SCI) occurs following different types of crushes. External and internal outcomes of SCI are including paralysis, cavity, and cyst formation. Effects of dietary derived antioxidants, such as palm vitamin E on central nervous system (CNS) encourage researchers to focus on the potential therapeutic benefits of antioxidant supplements. In the present study, experiments were carried out to evaluate the neuro-protective effect of the palm vitamin E on locomotor function and morphological damages induced SCI. Seventy-two male rats (Sprague-Dawley) were randomly divided into four groups: sham (laminectomy); control (supplemented with the palm vitamin E at a dose of 100 mg/kg/day); untreated-SCI (partial crush, 30–33% for 20 sec); treated-SCI (partial crush, 30–33% for 20 sec supplemented with the palm vitamin E at a dose of 100 mg/kg/day). The treatment with the palm vitamin E significantly improved the hind limb locomotor function, reduced the histopathological changes and the morphological damage in the spinal cord. Also, the palm vitamin E indicated a statistically significant decrease in the oxidative damage indicators, malondialdehyde (MDA) level and glutathione peroxidase (GPx) activity in the treated-SCI compared to the untreated-SCI.

## Introduction

Some major clinical health problems cause potential to immobilize a person suspected of having a spinal cord injury (SCI)^1^. Although many novel therapies are lunched to enhance neuroregeneration in SC after injury, but the rate of disability after SCI are still high^2^. The National Acute Spinal Cord Injury Study (NASCIS) reported in 1990 that the neurologic outcomes were improved by intra-venous (IV) of methylprednisolone (MPS) in those patients with SCI when they were administered within 24 hours: in the first 8 hours of the SCI, the highest dose of MPS was necessary^3^. Till now, some major side effects of MPS, such as depression, dizziness, anxiety, toxicity on kidney and liver have been reported^4,5^. Such problems encourage researchers to develop investigations for discovering new therapeutic agents with minimum side effects and high efficiency. Through human clinical trials conducted at the University of Maryland Medical Centre in 2004; a new drug namely mono-sialo-tetra-hexosyl-ganglioside 1 (GM1) antibody for improving of SCI has been proposed^5,6^. Those patients treated with GM1-ganglioside showed significant improvement in their neurologic recovery during the first year after the injury compared to those who were receiving placebo. These findings were based on the early animal model reports which suggested that GM1-ganglioside and MPS were effective drugs for improving the metabolic, vascular, functional, and pathological outcomes of traumatic SCI^5,6^. Antioxidant effects of palm vitamin E, increase the Basso, Beat-tie and Bresnahan (BBB)^7,8^ locomotor function scale of animals with SCI^9^. There are some reports which indicate that palm vitamin E deficiency may lead to pathologic changes in muscular and nervous systems^10,11^.

In a review study carried out by Valero in 2014^12^ on mitochondrial biogenesis, it was noticed that antioxidant could have a key role on reducing progressive tissue damage which could lead to improve recovery during post-traumatic SCI. Recent advances have surfaced that mitochondria, the organelles mostly known as the engine of cells, are also involved in many other cellular activities, such as lipid modifications, redox balance, calcium balance, and even cell death^13^. Nevertheless, mitochondrial dysfunction in the neural system could related to some neural disorders such as neuropathy, ataxia, mitochondrial encephalo-myopathy, and so on^14,15^.

Palm oil fatty acid is very well-known oily mixture among common vegetable oils in having a significant high content of tocotrienol. Tocotrienol extracted from crude palm oil is mainly composed of a mixture of alpha, gamma, and delta tocotrienol, which referred to as tocotrienol-rich fraction (TRF)^16^. At lower concentrations, TRF works as an antioxidant to regulate mitochondrial functions which were found to be associated with many pathologies, such as aging and neuro-degenerative diseases^17,18^.

There are many evidences illustrate that administration of alpha-tocopherol and alpha-tocotrienol could have possible neuroprotective effects on SCI due to its high antioxidant properties^19^. As a result, it could prevent the damage with subsequent recovery of both motor and sensory functions and improve the oxidative stress level with subsequent reduction of incidence of neurological deficits due to spinal cord conditions^20,21^. Such benefits for alpha-tocotrienol suggest that it is more significant compared to the alpha-tocopherol^22^.

In this study, by knowing that “supplementation with palm vitamin E is able to improve the recovery during SCI in animal models”, therefore, it is decided to evaluate the effect of the palm vitamin E in SCI by observing the body weight, locomotor function and morphological changes. For this purpose, the effect of palm vitamin E on the lipid peroxidation changing via measuring of the malondialdehyde (MDA) level and the activity of Glutathione peroxidase (GPx) following SCI is assessed for the first time.

## Results

### Changes in body weight

**Figure 1:**
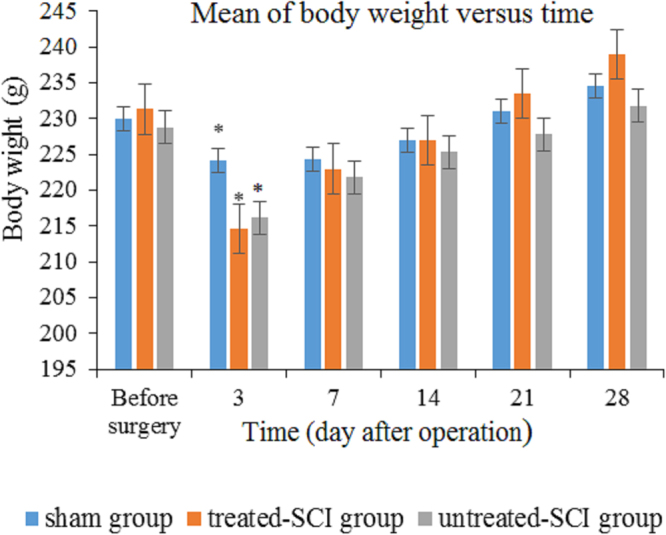
Changes in the body weight in rats at pre-test and post-operation time. *p < 0.05 considered to be significantly different values compared to sham group in the respective day.

The body weight began to decrease significantly in the treated-SCI, untreated-SCI, and sham groups within the first week after surgery compared to beginning of the surgery (250 ± 50 g) and then increased within the next 3 weeks. At the beginning of the fourth week, the rat’s weights restored to the initial weight. The body weight changes in the sham group were significantly faster compared to those treated-SCI and untreated-SCI groups (Fig. 1).

### Locomotor function assessment

**Figure 2:**
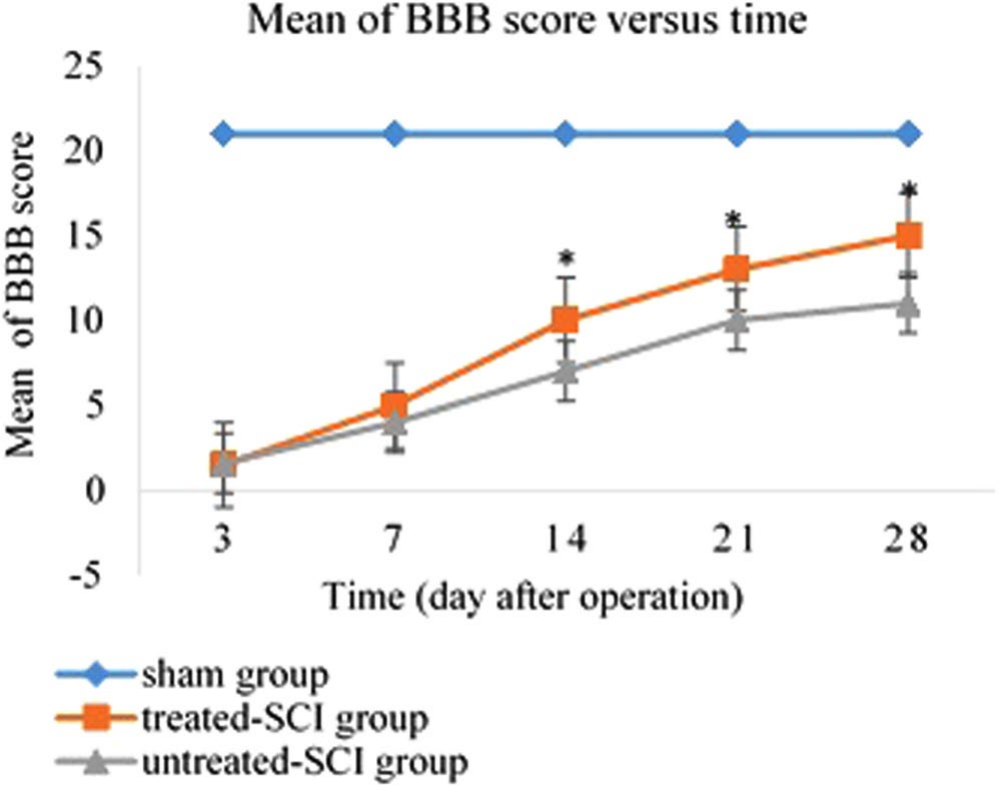
Behavioural improvement after palm vitamin E treatment. Locomotor functions were monitored by the BBB score among all the groups one day before (pre-test) and after operation. 28 days after treatment, the mean BBB score in the treated-SCI was significantly higher than that in the untreated-SCI group (mean ± SD, *p < 0.05) considered to be significantly different values versus sham group.

Locomotor skills were assessed by the open field test using the BBB rating scale. The BBB score was done on the day 1, 3, 7, 14, 21, and 28 post operation (PO). The sham group obtained the BBB score of 21 and there was no decline in the locomotor function of their hind limb within 28 days (Fig. 2). The difference in improvement of BBB score was measured significantly (P < 0.05) between the treated-SCI group compared to the untreated-SCI group at day 28. The BBB score of 0–1, which means acute flaccid paralysis (AFP), was observed in the treated-SCI and untreated-SCI groups with a little or no movement of the hind limb during the first week after the partial crush. Afterwards, an extensive or little movement in the hind limb joints were observed in the treated-SCI and untreated-SCI rats between week 1 to week 2. Some shortages of coordination were observed in the untreated-SCI group on the first day after surgery (BBB score 0–1). Throughout the first two weeks, the untreated-SCI group were found to be in a recovery position of their trunk and paw position, toe clearance, coordination, and also hind limb joint’s movements.

### Histopathology

**Figure 3:**
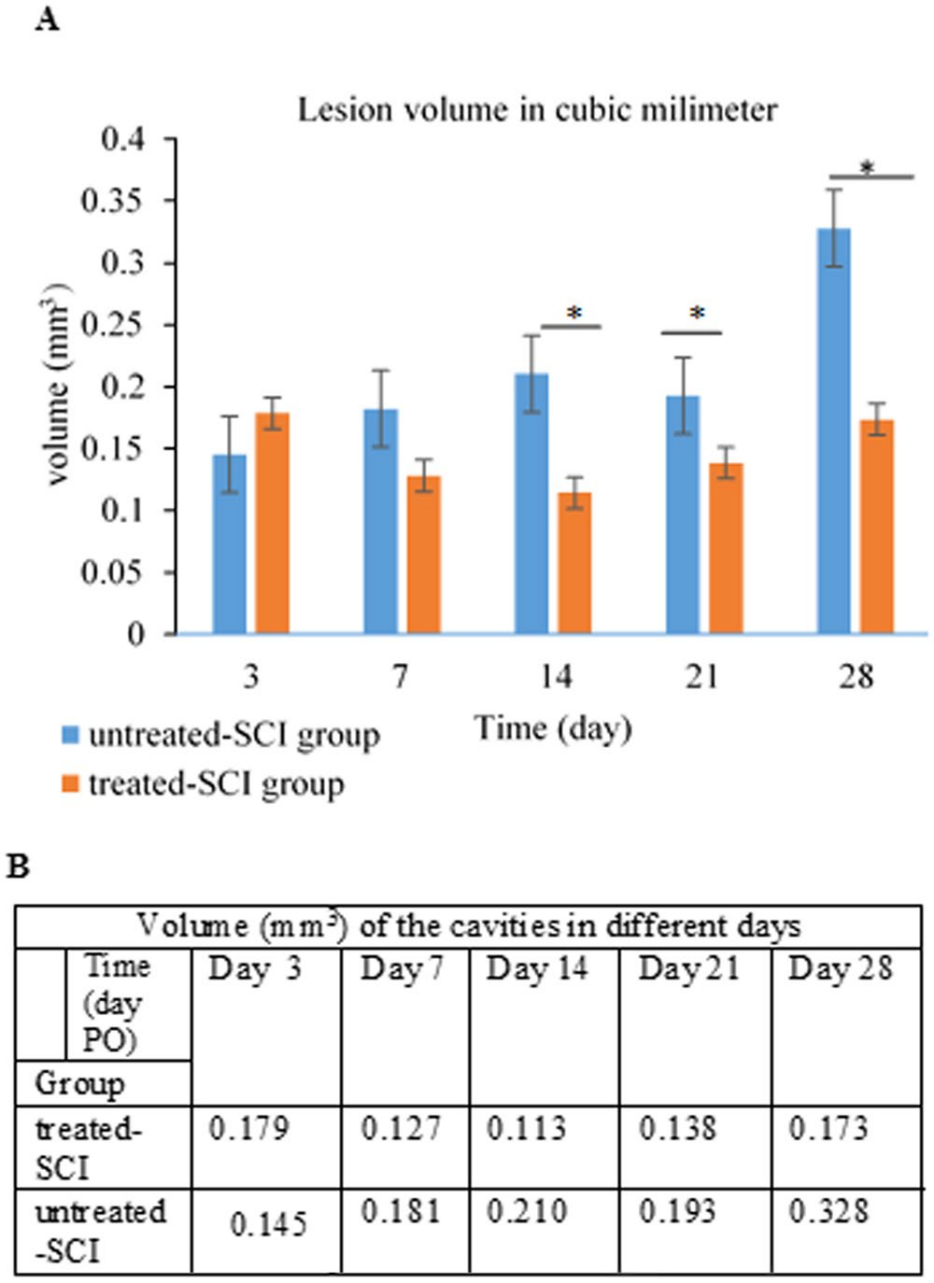
Reduced cavitation after palm vitamin E treatment. For measurements of the cavity volume, H and E sections were examined as detailed in material and methods. (**A**) Representative images of longitudinal sections of injured spinal cord 28 days after treatment. (**B**) Quantitative analysis of the cavity volumes on the basis of histological longitudinal images. The volume of the cavities in treated-SCI group decreased from day 3 to day 14 and then slightly increased within the rest days. In untreated-SCI group the volume of the cavity was slightly increased, then significantly increased in day 28. Data expressed as mean ± SD. *p < 0.05.

**Figure 4:**
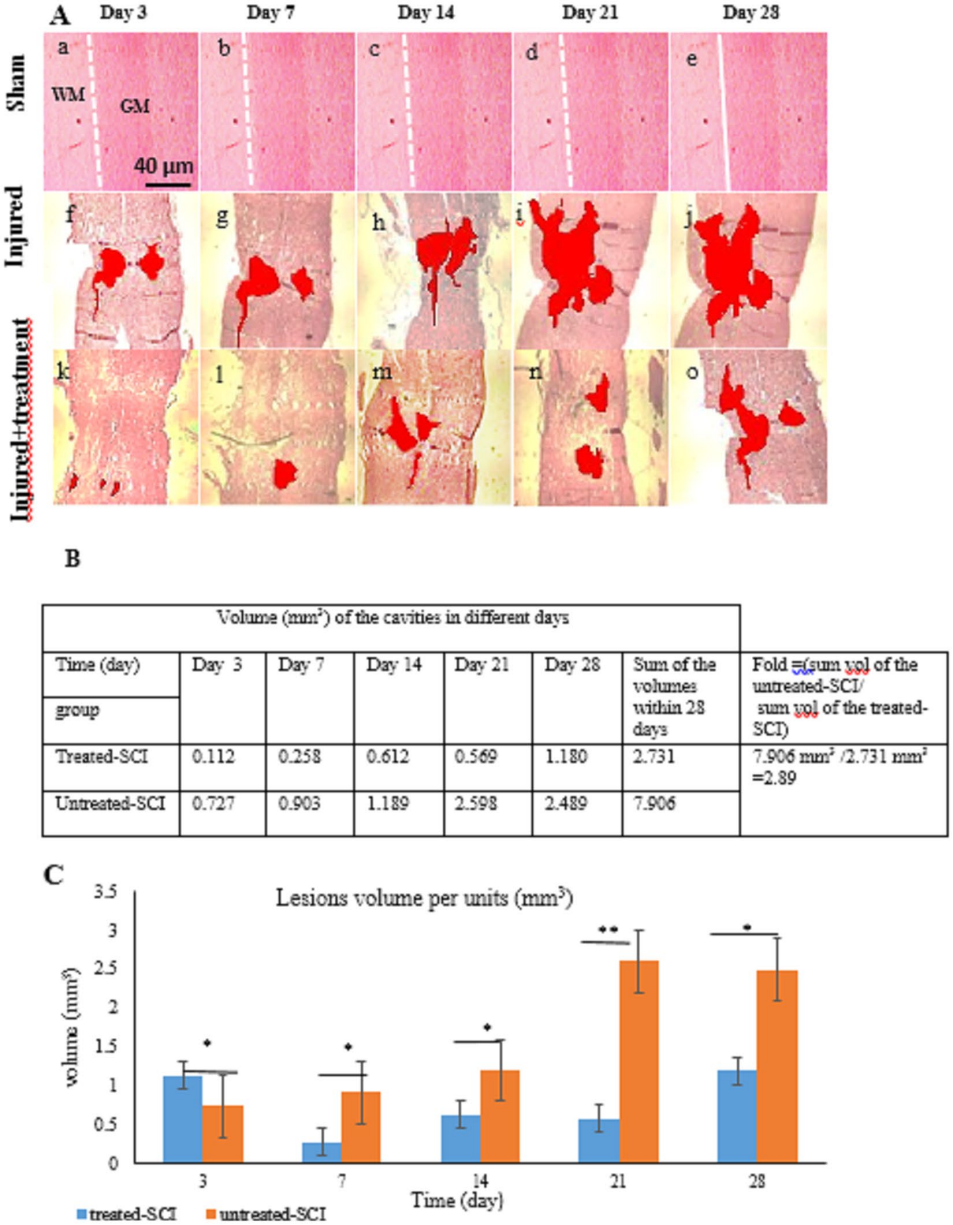
The effect of palm vitamin E on the lesion size and cavity volume of the spinal cord injured groups. (**A**) Microscopic observation of the spinal cord lesion area in the sham, treated-SCI and untreated-SCI groups at different time point has carried out by H and E staining. (a–e) The microscopic intact grey matter and white matter in the sham group after laminectomy at different time point. The sham group spinal cord in the all times of study is intact. (f–j) The lesion area and the cavity formation shaded at study days in the untreated-SCI group. (k–o) The lesion area and cavity shaded in the treated-SCI group. The shadow area shows cavity volume in lesion part of spinal cord crush injured rats. (**B**) Quantitative analysis of the cavity volumes on the basis of histological longitudinal images shows significantly increased cavity volumes in the untreated-SCI group compared to the treated-SCI group. (**C**) Different cavity volume between the treated-SCI and untreated-SCI groups. GM; grey matter, WM; white matter, BL; border line between grey matter and white matter. Scale bar, 500 μm for panel (a) to (o), under ×4 magnification. Data expressed as mean ± SD. *p < 0.05, **p < 0.01.

The untreated-SCI and treated-SCI were sacrificed on day 3, 7, 14, 21 and 28 PO. The cavity formation could be observed from day 7 to 14 which followed by the cyst formation after the serial longitudinal sectioning. In the microscopic assessment, the cavity volume size was decreased between the day 3 (0.1788 ± 0.04 mm^3^) to day 14 (0.1142 ± 0.08 mm^3^) in the treated-SCI group and then inclined between day 14–28 was slightly from 0.1142 ± 0.1 mm^3^ to 0.1735 ± 0.04 mm^3^. While in the untreated-SCI group, the graph depicts a slight increase at day 3–14 (0.1454 ± o.1 to 0.2103 ± 0.08 mm^3^) and then sharply increased to day 28 (0.3277 ± 0.06 mm^3^) (Fig. 3A,B). The identification of the haemorrhage areas was possible in the lesion epicentre instantaneously after SCI (Fig. 4A) which resulted in increasing of the SC haemorrhage. Also, the cavity formation and oedema were observed within first week after the injury. As it is listed in Table 1, there were significance (p < 0.05) differences of the lesions size between the rostral-to-caudal and lateral-to- lateral in the treated-SCI and untreated-SCI groups at any point of the time. The lesion volume sizes were determined by lateral-to-lateral, rostral-to-caudal and specimen thickness (5 µm) measurement (Fig. 4A). Using Image J software, the yielded sums of the volume size in the treated-SCI group and untreated-SCI group were 2.731 mm^3^ and 7.906 mm^3^, respectively. As shown in Fig. 4B, the untreated-SCI group cavity volume size was 2.89 times higher than that for the treated-SCI group. Due to different times of studies, the measurements of the size cavity were not the same. Figure 4C depicts the sums of volume cavity sizes of the treated-SCI and untreated-SCI groups.

**Table 1:** Measurement of the rostral-to-caudal and lateral-to-lateral of the cavities in the treated-SCI and untreated-SCI groups within 28 days after spinal cord injury.

Cavity formation measurement										
rostral to caudal cavity (µm)						lateral to lateral cavity (µm)				
Day (PO)	3	7	14	21	28	3	7	14	21	28
Treated-SCI group	1476.93 ± 2.2	1728.64 ± 2.1	1232.50 ± 3.3	1386.41 ± 1.2	1787.13 ± 3.1	1210.83 ± 3.2	1484.38 ± 2.9	1864.37 ± 3.2	1998.20 ± 1.8	1942.00 ± 3.9
Untreated-SCI group	1793.24 ± 1.3	1678.08 ± 1.0	1757.92 ± 0.2	2314.47 ± 2.2	2285.36 ± 2.8	1622.60 ± 3.2	2173.48 ± 1.5	2394.09 ± 1.7	2600.14 ± 2.6	2868.25 ± 2.6

Data are expressed as mean ± SD. PO: post operation.

**Figure 5:**
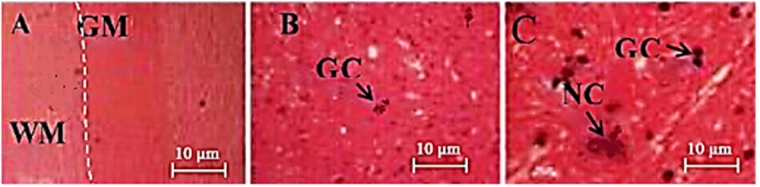
Sections of white matter and grey matters spinal cord of the animals subjected to laminectomy as seen by haematoxylin and eosin (H and E) staining. (**A**) Normal structure of the grey and white matter in the sham group rats. (**B** and **C**) The sham group shows the normal spinal cord parenchyma with the normal-appearing neural cell and glial cell. (**C**) Normal neural cell with nucleus approximately in the centre of body cell. The distributions of cell organelle are clearly visible. Scale bar, 500 μm for (**A**) Under x4 magnification; 100 μm for (**B**) Under x40 magnification and (**C**)10 µm, under x100 magnification. NC, neural cell; GM, grey matter; WM, white matter; GC, glial cell; BL, border line between grey matter and white mater.

**Figure 6:**
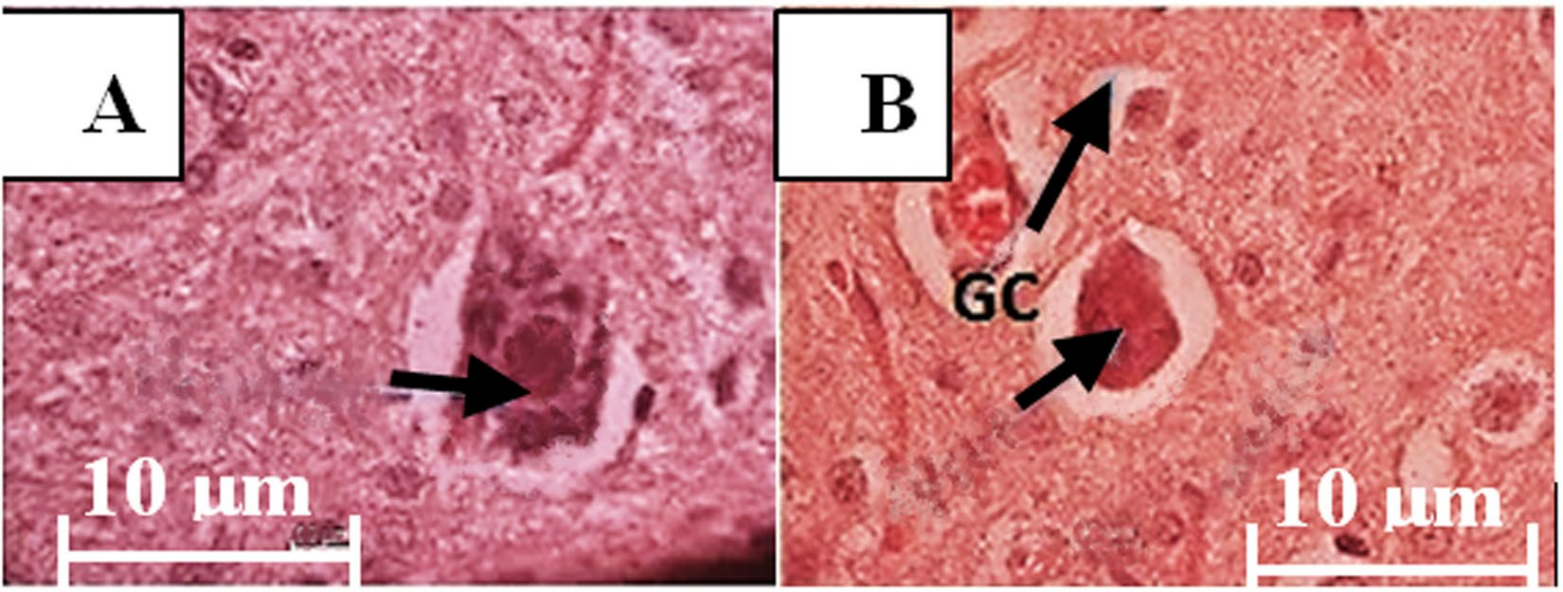
Microscopic H and E observation in the treated-SCI and untreated-SCI groups. (**A**) The normal neural cells in high concentration of cytoplasmic body in the treated-SCI group at day 21 are observed, and the nucleus is placed in the centre of the neural bodies. (**B**) In the untreated-SCI group, a few erythrocytes beside the unformatted neural cell with glia cells are seen at day 21. Inside the neural cell is without concentration of cytoplasmic body and nucleus start enlarging leading to be necrosis. Scale bar 10 μm, x100 magnification; N: nucleus; GC: glial cell.

In the sham group, there were no hind limb locomotor dysfunction immediately after anaesthesia till to the end of the study (day 28). Also, in the microscopic observation, the intact grey and white matters with distinct border lines were found (Fig. 5). In the present study, another side microscopic observation showed agglomeration of the erythrocyte in the cavity-shape area at day 3 in the treated-SCI group, which was much higher compared to those observed in the treated-SCI group. Till day 21, in the untreated-SCI group, abnormal neural cells with pale nucleus, more glial cells and also some cavities could be observed (Fig. 6).

### Lipid peroxidation (MDA) analysis

**Figure 7:**
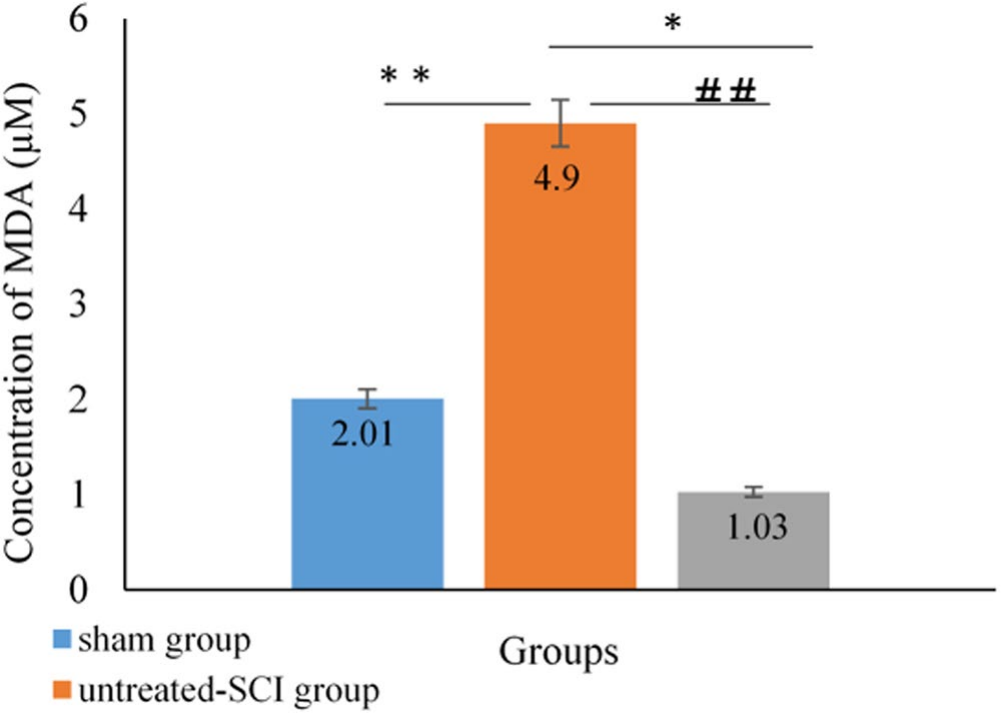
Effect of palm vitamin E on the tissue MDA level after spinal cord injury. Data are expressed as mean ± SD. Means among groups (n = 6 rats/group) show significant difference, **P < 0.01 and *P < 0.05 compared to the sham group, and ^##^p < 0.0 1 compared to the treated-SCI group.

A statistical significant (P < 0.05) difference of the tissue MDA levels which were calculated using the protocol inside the kit was observed in the untreated-SCI, treated-SCI and sham group at all points of time. The MDA level was found much higher in the untreated-SCI (4.9 µm) compared to that in the treated-SCI (1.2 ± 0.06 µm) and sham (2.1 ± 0.00 µm) groups (Fig. 7).

### Tissue Glutathione Peroxidase (GPx) analysis

**Figure 8:**
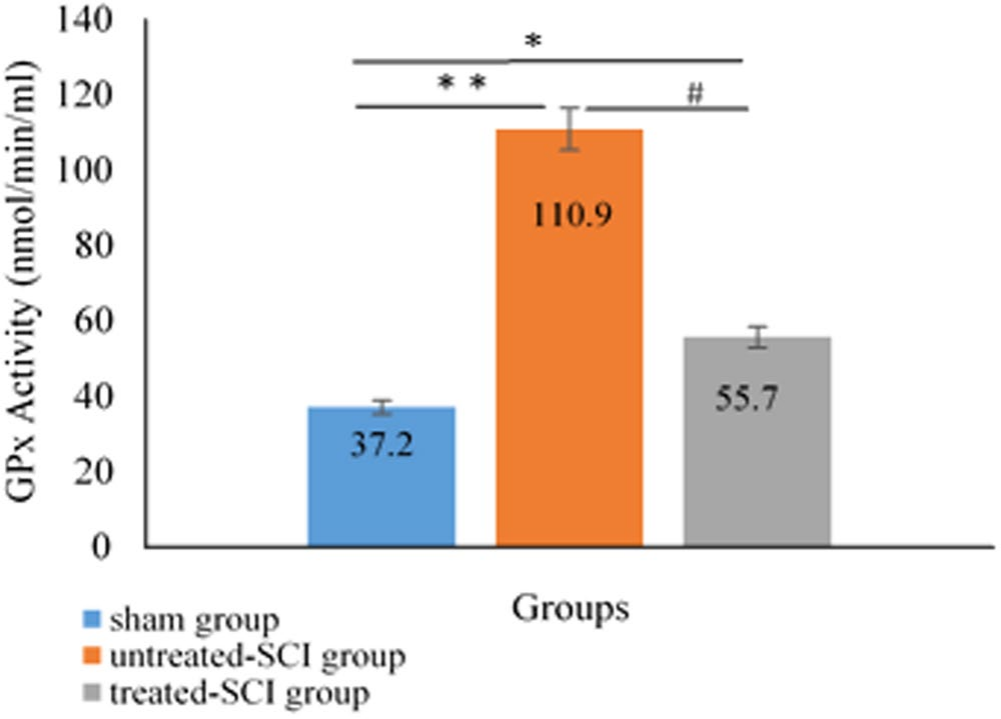
Effects of palm vitamin E on the level of the GPx activity level after spinal cord injury. Data are expressed as mean ± SD. Means among groups (n = 6 rate/group) show significant difference, **P < 0.01 and *P < 0.05 compared to the sham group and ^#^p < 0.0 5 compared to the treated-SCI group.

The tissue GPx activity was found markedly different in the treated-SCI, untreated-SCI and sham groups. The GPx activity increased in both treated-SCI (57 ± 1.5 nm/ml) and untreated-SCI (111 ± 3.2 nm/ml) animals compared to that in the sham (38 ± 1.5 nm/ml) group. The spinal cord antioxidant marker, such as GPx concentration elevation, could be a sign to of oxidative stress caused by injury which will be discussed further in the paper (Fig. 8).

## Discussions

In the present study, the oral supplementation of palm vitamin E at a dose of 100 mg/kg/day reduced the neurological deficits in the spinal cord injury. This dose is almost equivalent to the normal human dose (50–200 mg/day). Published studies have proved that palm vitamin E at a dose of 100 mg/kg showed neuro-protective and also neuro-regenerative activity due to its antioxidant properties^19,21–24^.

Our data showed the treated-SCI rats displayed less cavity formation, suggesting that palm vitamin E possessed anti-trauma and antioxidant properties which are in parallel to those of pervious findings^25,26^. The method used in current study for causing the crush injury, which led to the trauma, was the application of Inox no. 2 forceps. The reason of applying this method is because only a forceps is required which is cheap and easy to use. Therefore, it is introduced as a more effective candidate than other methods, such as weight dropping or balloon compression^27,28^.

Although the body weight changes were stable throughout week 3 to 4 PO, but a slight drop in weight was observed during the first week after the surgery. Some factors such as stress, SC trauma, and loss of blood or fluid during the surgical procedure are being suggested which could be related to the weight loss^29^. Another word, the main important factors causing weight lost through the first week was highly suggested due to the rat’s hind limb locomotor dysfunction or paralysis and also metabolic parameters affected by the operation. The pathophysiological implications of such dysfunctions were represented by the increase in production of the reactive species which are extremely aggressive to the surrounding tissues^30^.

In current research, the treated-SCI group showed gain weight compared to the untreated-SCI group which might be due to the antioxidant effect of the palm vitamin E. Bedreag *et al*., in 2014^30^ showed that ill patients with spinal cord injury were more prone to oxidative stress due to the multitude of the biological dysfunctions. There are many biologically active substances with antioxidant properties of palm vitamin E that have proved to be able to reduce oxidative stress which contribute to boost up the recovery after SCI^29^. The preserved body weight in the sham group was associated with absence of any operation on the spinal cord and also having sufficient nutrition^31,32^. Since, the spinal cord crush limited access to enough food or water, therefore, the inconsistency in the weight loss was observed^32^.

We found that the BBB score varies on day 3, 7, 14, 21, and 28 PO in the untreated-SCI, treated-SCI, and sham groups. In the sham group, only the vertebrae of thoracic area were removed, while the SC was completely intact. Thus, the animals with no damage in the spinal cord did not show any changes in their BBB score during 28 days. It was reported that laboratory animals with incomplete contusion SCI reached maximal locomotor recovery within the first few weeks’ post-injury^33,34^. So, the SC possessed the main role in performing locomotor tasks. There are some evidences to suggest that SC trauma, except the external signs and symptoms, was dependent to several plasticity caused contusion, hemi-contusion, hemi-section and etc.^33,34^. The results of our current study showed that there were no changes in the BBB score in the treated-SCI and untreated-SCI groups during the first 7 days. Afterward, the different BBB score in the named groups started to increase significantly, while on day 28 the BBB score for the treated-SCI group reached a score of 15 but the untreated-SCI group gained a score of 11. It is found that the palm vitamin E having potent antioxidant properties is able to exert protective effects by the reduction of oxidative damage. After SCI, a variety of oxygen radicals released from the cells which led to cell damages. The free radicals were scavenged by the palm vitamin E which contributed to reduction of the cell damages resulting in improvement of the locomotor function^35^. The main behavioural differences between the treated-SCI and untreated-SCI groups could be anticipated as the extensive movement of all three hind limbs joints (ankle, knee and hip) which were observed in all the studied animals. Although, various methods such as graded hemi-contusion, contusion, hemi-section etc^31,33^. have been applied to evaluate locomotor function in cervical and thoracic parts of spinal cord, but almost similar results in locomotor function have been reported^27,36^.

A large variety of semi-quantitative and whole scale based on the locomotor response of rats are currently being used to evaluate locomotor rating scale after SCI, such as BBB Locomotor Rating Scale^7,8^, 5-pt Tarlov Scale^37^ grid-walk test^38^, narrow beam test^39^ and inclined plane test^40^. It is claimed that those SCI rats which display alternative locomotor functions following injury could not be classified by qualitative visual based analysis. Therefore, many quantitative procedures were extended to evaluate SCI motor function^41,42^. Guertin in 2005^43^ recommended a new semi-quantitative assessment method called average combined scores (ACOS) which calculated basic locomotor-like activities based on the spinal reformation at the sub-lesion level. Compared to the BBB scale, the ACOS method was more multifaceted, time- and energy-consuming. ACOS uses video and electromyogram (EMG) recording to estimate locomotor recovery function which was more regarded as scientific quantitative assessment.

The various lesions of the rostral-to-caudal and lateral-to-lateral cavity sizes per time points are listed in Table 1. The lateral-to-lateral, rostral-to-caudal and specimen thickness measurements determined the cavity and/or lesion volume size per unit. There were significant changes in the cavity volumes and lesions size between the treated-SCI and untreated-SCI during the period of study, which could be due to the neurodegenerative effects and antioxidant activity of palm vitamin E.

Alpha-tocopherol and alpha-tocotrienol act as membranous antioxidants in mouse fibroblast cells^22^. In our study, the improvement of the BBB score in the treated-SCI group might be explained by the regeneration of the SC tissues affected by palm vitamin E which was also alluded in the literatures for organs such as, heart and cardio-vascular system, liver and coagulation system, brain and CNS, blood brain barrier and etc.^11^.

The study performed by Girard S, *et al*.^44^ in utilizing multiple behavioural tests for evaluating functional recovery and residual function after SCI in the rodent models supports our findings^45^. It is obvious that different level of SC trauma is considered as an important factor for developing future therapeutic approaches.

On the microscopic assessments during all the intervals post-injury, the cavity lesions were smaller in the treated-SCI group compared to the untreated-SCI rats. Since palm vitamin E acts as a potent antioxidant agent in mitochondrial injury recovering, therefore, it might improve survivability upon glutamate toxicity due to its capability of oxidative stress elevation^10,46,47^. Researchers are using different types of vitamin E, such as tocotrienol-rich fraction (TRF) or alpha-tocopherol for treatment of different tissue damages. Xueling *et al*.^19^ showed that TRF could have better curing effects on different tissue abnormalities compared to tocopherol types.

Antiserum inhibitor of nitric oxide synthesis^12^, Nitro steroid NCX 1015^48^ intra-peritoneal (IP) citocholine^49^ and Curcumin^50^ because of their antioxidant effect, are able to reduce SCI following by trauma. However, many promising attempts have been made to reduce the signs of SCI, but none have yielded reliable effects.

It is suggested that free radicals and subsequent lipid peroxidation may delay the treatment progress of SCI-induced by any type of trauma. Antioxidants such as those from palm vitamin E family reduce trauma after SCI. Thus, motor dysfunction after SCI is shown to be affected by alpha-tocopherol^51,52^.

The MDA levels increased in the untreated-SCI, while the palm vitamin E supplementation delayed this process in the treated-SCI group. literature study showed that treatment with palm vitamin E attenuated the IL-1β in the injured SC, and also decreased the oxidative stress infiltration of neutrophils after SCI^52^. We evaluated the therapeutic effects of the palm vitamin E on the SC specimen using spectrophotometer and noticed that serious neuronal loss diffuse haemorrhagic areas were accumulated in SCI animals at 24 hours after operation. It was suggested by Miriam *et al*.^53^ that IL-1β played a harmful role in the progress of post-traumatic damage. Such effects could be reduced by blocking its signalling pathways. We followed some of the probable mechanisms by which the palm vitamin E attenuated neurological damage. It was shown that SCI enhanced penetration of oxidative stress, which was determined by an increase in lipid peroxides and diminution of enzymatic (GPx) antioxidants in the tissue. The palm vitamin E exerted dominant antioxidant effect in conjugation with a decrease of the free radical-derived products (MDA), and improved the recovery in SC or CNS after injury^54^. In the untreated-SCI group, an increase in the free radical activity was observed which in turn could enhance the levels of the MDA and GPx. In the treated-SCI group, a significant decrease was observed in the antioxidant activity compared to the untreated-SCI and sham groups which was in response to the oxidative stress followed by injury. This findings are supported by the work reported in the literature that palm vitamin E dietary supplementation showed brain and neuronal cells protective effect against dysfunction brain injury and Alzheimer disease^19,55^.

## Conclusion

It is concluded *in vivo* studies that on 28 days of the treatment of SCI with the palm vitamin E could have the maximum reduction in spinal cord cavity and cyst formation. Four weeks after the treatment, an increase regenerating in spinal cord is observed leading to functional recovery of the essentially paralyzed hind limbs. The spinal cord injury lead to paralysis, mostly to the hind limb. It is found that the palm vitamin E is a promising impact on the injured spinal cord. These beneficial effects, together with the biological safety and other properties such as antioxidant effect suggests that this approach may have clinical applications in the treatment of SCI.

## Methods and Materials

### Animals

Eight-week-old male Sprague-Dawley rats weighing 250 ± 50 g were used (n = 72). The animals were housed in an environment with a 12 hours’ dark/light phase at the standard condition of temperature (21 ± 1 °C) and controlled humidity (50 ± 10%). The standard laboratory food and water were given *ad libitum* and each wire cage housed two rats. The experimental processes in this study were approved by the Animal Care Committee of the Faculty of Medicine’s Animal House, Institutional Animal Care and Use Committee; University Malaya (UM), according to the guideline and policy of University Malaya; regarding the care and use of animals for scientific purposes with the reference number.

Seventy-two Sprague-Dawley (SD) rats were blindly and randomly divided into four groups: Sham (laminectomy alone, n = 6); control (intact animals supplemented with the palm vitamin E; at a dose of 100 mg/kg/day; 28 day, n = 6)); untreated-SCI (partial (mild) crush 30–33% for 20 sec, n = 30); treated-SCI (partial (mild) crush 30–33% for 20 sec, supplemented with the palm vitamin E; at a dose of 100 mg/kg/day; 28 day, n = 30).

The number of animals was calculated using Power analysis based on the pervious study from our lab^56^.

### Surgical procedures

The rats were anesthetized using a mixture of 100 mg/kg of kertamil (100 mg/ml ketamine, Australia) and 10 mg/kg of xylazil (20 mg/ml xylazine, Australia) through intra-muscular (IM) injection. 70% alcohol was used to clean the skin and then, the back thoracic area were shaved for further experiments. The spinal column was exposed to T11-L1 and, for the transverse apophyses, the paravertebral muscles were removed. For the sham group, the SC was opened but no injury was inflicted, and for the treated-SCI and untreated-SCI groups, to manage the partial crush injury, Inox no.2 modified stopper forceps were used. The partial crush was administered at the T12 level of the spinal cord. The saline solution was applied to clean the wound and then for repair the fascia, muscles, and skin, the absorbable sutures and clips were used, respectively. During surgery, the body temperature was maintained at 37 ± 0.5 °C constantly, while a hot plate was applied to preserve the same temperature during the post-operative (PO) period^57^.

### Post-injury Care

After the operation, a 1 ml of the lactated ringers’ solution was injected subcutaneously (SC), and the topical antibiotic was applied to the skin at the incision site. A 5% Baytril (Bayer, Korea) IP injection was given at a dose of 2 mg/kg/day for one week after the surgery. After the operation, an analgesic was administered as well as it needed (Buprenorphine 5 µg/kg every eight hours). The food was always accessible in the food pellets were placed on the bottom of each cage. The gastrointestinal and hydration functions were monitored every day. During one week after surgery, the rats were weighed every day; after which they were weighed every week. Post-operative care comprised manual bladder expression twice a day until the bladder function returned.

### Palm vitamin E and diets supplementation

The four groups were fed with a standard rat chow. The animals were given access to food and water *ad libitum*. The control and treated-SCI groups were supplemented with 100 mg/kg/day palm vitamin E (through an oesophageal feeding tube) for 28 day according to the protocol published by Yao *et al*.^58^.

### Behavioural analysis

#### Open field task

At pre-test time (one day before surgery), the rats underwent locomotor function evaluation in the experimental chamber. During pre-test evaluation, all animals environmental condition such as temperature, humidity and light, should be stable similar to experiment days. Each animal’s locomotor activity in both pre-test and experiment time was evaluated by two examiners in an open field system over four minutes. The open field was a plexi-glass circle chamber with 100 × 100 cm dimensions and the walls measuring about 9–11 cm, placed at a room temperature with normal lighting and a quiet environment.

### Hind limb locomotor function

The rats were also assessed 1 and 3 days after surgery, after which their assessment was carried out once a week until day 28 after the operation. The certain functional behavioural conditions were measured using the BBB locomotor rating scale. This scoring system is explained by Basso *et al*.^8^. The specific components of functional behaviour were assessed such as tail position, trunk and abdomen position, coordination, toe clearance, stepping, limb mobility, and paw position. In the BBB scale, a score of 21 is an indication of normal hind limb locomotor function (complete mobility) and a score of 0 is a sign of lack of natural hind limb movement (complete immobility).

### Histopathological assessment

#### Tissue processing

At the time of sacrifice, a mixture of xylazine (0.3 mg/kg, xylazine, Australia) and ketamine (0.7 mg/kg ketamine, Australia) were used to deeply anaesthetize the rats via IM. Each rat was put in their prone position and the thorax was shaved. Using scissors to cut both sides of the sternum, the cardiac area was opened and the left ventricle (cardiac apex) was perfused using a butterfly angiocath perfusion catheter. The vena cava or left atrium were cut and the area perfused with about 50 ml normal saline (until/clear). This was followed by about 150 ml of 4% formalin in millioning’s phosphate buffer, applied at the rate of 350–400 ml/min for the peristaltic pump. The vertebrae were then removed and placed in 4% formalin in millioning’s phosphate buffer overnight at an approximate temperature of 4 °C, constantly and then in the following day, the spinal cord was removed from the vertebrae.

### Haematoxylin and Eosin staining

To prepare the serial sections (5 µm), a section (1 cm) from rostral-to-caudal of the lesion epicentre was placed in paraffin in tissue-Tek; tissue embedding machine with laboratory cryostat. Haematoxylin and Eosin staining (H and E) and light microscopy were used to carry out the histopathological evaluation of the SCI for each sample. Then the cavity area for the lesion of the sham, treated-SCI and untreated-SCI group, traced and then calculated.

### Lesion volume estimation

The lesion/cavity size in the treated-SCI and untreated-SCI groups was estimated using the open source, ImageJ analysis software (NIH; National Institutes of Health download: http://rsb.info.nih.gov/ij/) version of 1.47 v^59^ as per directions of the ImageJ developers. The image analysis was performed on the digital photos manipulated by NIS system. In longitudinal serial sections containing a central lesion area of the cavity formation was shaded and then determined. The total area of the cavity (lateral-to-lateral and rostral-to-caudal of each lesion) was recorded in an equal thickness (5 µm) of each sections. The total longitudinal-section of the volume lesion area of the spinal cord, were calculated in the treated-SCI and untreated-SCI groups at each time point of study^60^.

### Quantitative image analysis procedure

The photographs of the spinal cord specimens were placed under the microscope (Nikon, Japan) and then analysed. The injury border was drawn, and the lesion area of rostral-to-caudal, lateral-to-lateral cavity and cyst formation measurement were calculated for each sample using the NIS-Elements AR 3.2 software connected to the microscope eclipse (Nikon eclipse 80*i*, Japan). In the treated-SCI and untreated-SCI groups, the area of the lesion was discerned by the presence of necrosis, inflammatory cells, cavity and also cyst formations. The lesions sizes were measured according to two parameters: rostral-to-caudal and lateral-to-lateral in cavity and cyst formation.

### Lipid peroxidation (MDA) analysis

MDA is a natural product of lipid peroxidation. Lipid peroxidation is a well-known mechanism in animal and plants cell damage. It is also used to suggest oxidative stress in cells and tissues^61^. The SC specimen from the sham group, only in area of SC which removed vertebrae and in treated-SCI and untreated-SCI groups at the injured area (1 cm rostral and 1 cm caudal from lesion area) were removed 24 hours after scarify of rat at day 28 and immediately stored at −80 °C until use. The specimens were homogenized separately in the cold assay buffer (on ice) and then centrifuged at 12,000 rpm for 30 min at 4 °C. MDA level was assessed with the thiobarbituric acid reactive substances TBARS (TCA Method) assay kit; Cayman chemical, USA, item No. 700870) according to the manufacturer’s instructions.

### Glutathione Peroxidase Assay (GPx)

Glutathione peroxidase (GPx) catalyses reduction of hydro-peroxides including hydrogen peroxide, by decreasing glutathione function to protect cell from damage. The spinal cord specimen from the sham, treated-SCI and untreated-SCI groups, 24 hours after scarify of rat at day 28, were removed and homogenized separately in 5 volumes of the cold assay buffer (50 mM Tris-HCL, pH 7.6, containing 5 mM EDTA)/g)^62^. The homogenised specimen was then centrifuged for 15 min at 10000 g at 4 °C and the supernatant was removed for GPx according to the manufacturer’s protocol (Cayman chemical, USA, item No. 703102).

### Statistical analysis

The data were analysed using SPSS 11 for Windows. All outcomes are expressed as means ± SD. One-way analysis of variance (ANOVA) with Tukey’s post hoc analysis was used to compare differences among the groups. If the P value was less than 0.05, it was considered statistically significant. The data collected from the experiments was recorded and analysed using SPSS software (version 19.0, Inc., Chicago, IL, USA).
